# Evaluation of tomotherapy MVCT image enhancement program for tumor volume delineation

**DOI:** 10.1120/jacmp.v12i3.3505

**Published:** 2011-04-12

**Authors:** Spencer Martin, George Rodrigues, Quan Chen, Simon Pavamani, Nancy Read, Belal Ahmad, J. Alex Hammond, Varagur Venkatesan, James Renaud, Slav Yartsev

**Affiliations:** ^1^ Department of Physics London Regional Cancer Program London Health Sciences Centre; ^2^ Department of Radiation Oncology London Regional Cancer Program London Health Sciences Centre; ^3^ Department of Physics and Astronomy University of Western Ontario London Ontario Canada; ^4^ Department of Oncology University of Western Ontario London Ontario Canada; ^5^ Department of Epidemiology and Biostatistics University of Western Ontario London Ontario Canada; ^6^ TomoTherapy Inc. Madison Wisconsin USA

**Keywords:** tomotherapy, MVCT enhancement, target delineation

## Abstract

The aims of this study were to investigate the variability between physicians in delineation of head and neck tumors on original tomotherapy megavoltage CT (MVCT) studies and corresponding software enhanced MVCT images, and to establish an optimal approach for evaluation of image improvement. Five physicians contoured the gross tumor volume (GTV) for three head and neck cancer patients on 34 original and enhanced MVCT studies. Variation between original and enhanced MVCT studies was quantified by DICE coefficient and the coefficient of variance. Based on volume of agreement between physicians, higher correlation in terms of average DICE coefficients was observed in GTV delineation for enhanced MVCT for patients 1, 2, and 3 by 15%, 3%, and 7%, respectively, while delineation variance among physicians was reduced using enhanced MVCT for 12 of 17 weekly image studies. Enhanced MVCT provides advantages in reduction of variance among physicians in delineation of the GTV. Agreement on contouring by the same physician on both original and enhanced MVCT was equally high.

PACS numbers: 87.57.N‐, 87.57.np, 87.57.nt

## I. INTRODUCTION

CT‐based conformal radiotherapy aims to deliver therapeutic doses to target volumes while staying within the known tolerance of adjacent critical structures. Anatomical changes during the course of radiotherapy can result in underdosage to the target volume which may lead to disease recurrence and/or overdosing of healthy tissue with increased risk of complications. Image guidance provided by advantages in technologies such as cone‐beam CT and megavoltage CT (MVCT) offers a possibility for daily imaging, enabling clinicians to monitor anatomical changes during the course of treatment. The reliability of these images, however, is subjected to the interpretation of the contouring physicians and therapists. Errors in the delineation of the target volume can have an unfavorable impact on the success of the radiation therapy treatment and may contribute to subsequent side effects. Previous studies have reported the significant variability between clinicians in the delineation of gross tumor volumes for disease sites such as non‐small cell lung,^(^ ^1^ ^,^ ^2^ ^)^ prostate,^(^ ^3^ ^,^ ^4^ ^)^ and head and neck cancers.^(^ ^5^ ^,^ ^6^ ^)^ In the literature review by Weiss and Hess,^(7)^ head and neck tumor delineation experienced some of the highest variability between observers of any anatomy. Njeh^(8)^ stated that this can be attributed to many factors including imaging modality, contouring and imaging technique, and the influence of the observer. In addition, patients treated with radiation for head and neck cancers commonly undergo changes in the irradiated volume during radiation therapy. These changes, due primarily to radiation response, include reduction in the size of the tumor and enlarged neck nodes, as well as changes in the irradiated tissues due to weight loss and local changes such as postoperative edema.

Presently, planned adaptive software (TomoTherapy Inc, Madison WI) is used to analyze volume changes in patients via MVCT images acquired for daily setup verification purposes for patients undergoing radiation therapy with helical tomotherapy (HT) (TomoTherapy Inc, Madison WI). However, the quality of MVCT images acquired for head and neck patients is not ideal for unambiguous determination of anatomy, as indicated in previous publications.^(9)^ This study aims to compare the delineation of the GTV by the physicians of the head and neck team at our institution for original and enhanced MVCT studies. The need for higher quality onboard 3D‐MVCT imaging capability of HT stems from its use as the planning CT image acquisition method in both adaptive planning^(10)^ and the StatRT option (TomoTherapy Inc, Madison WI) of helical tomotherapy.^(11)^ The head and neck area is one of the most challenging sites for target contouring in radiation therapy, and the use of enhanced MVCT images could provide benefits, such as better locoregional control and the reduction of radiation related acute toxicity and late side effects caused by inaccurate target delineation for MVCT‐based treatment planning. In addition, daily treatment verifications require high quality MVCT images for reliable patient verification dose calculations.^(12)^ TomoTherapy Inc. is developing an MVCT imaging enhancement tool, (ImageTool, TomoTherapy Inc., Madison, WI) to improve the quality of MVCT images and, in this study, we report our results based on work done with this software.

## II. MATERIALS AND METHODS

Helical tomotherapy is a modality for delivering intensity‐modulated radiation therapy (IMRT) treatments using a linear accelerator mounted on a continuously rotating slip ring gantry. This technique combines the geometry of a diagnostic CT scanner with the capability to deliver highly conformal dose using 6 MV X‐ray beams with a collimated 40 cm wide fan of thicknesses 0.5 to 5.0 cm to an isocenter 85 cm away.^(^ ^13^ ^–^ ^15^ ^)^ Using the same X‐ray source, operating at 3.5 MV, megavoltage CT (MVCT) studies can be acquired prior to treatments of patients for daily registration with the planning kVCT image allowing for correction of patient setup.^(16)^ The Image Tool implements a tensor‐based anisotropic diffusion method^(^ ^17^ ^,^ ^18^ ^)^ that incorporates the directionality in the image analysis to better represent the 3D behavior of the edges and thus increase feature preservation. The trial version used in this study provides the user with three adjustable parameters to control the enhancement process. ‘Noise amplitude’ (adjustable from 0 to 100 HU) determines how much contrast is enough for image features to be preserved in the enhanced image by applying a Gaussian smoothing function to get rid of noise influence. This parameter regulates how distinguishable an object is from other objects and the background by comparing pixel values within a given image to adjacent voxels. If the difference is smaller than the number of HU specified by the user, the image feature is determined to be noisy and it is smoothed out. If the contrast difference is larger than the specified HU value, the image feature is preserved within the enhanced image. The ‘feature granularity’ parameter (adjustable in the range 0–3.5 cm) dictates how big in size the image feature should be to remain visible in the enhanced image, and allows us to avoid smoothing smaller, high‐contrast image features in the image enhancement process. Using a Gaussian smoothing function to eliminate noise‐related image features allows for the possibility of losing features in the anisotropic diffusion of the image that may be smaller than the Gaussian smoothing kernel size. The ‘Iterations’ option determines the number of optimization cycles the program will perform upon the image.

Five radiation oncologists (NR, SP, BA, JAH and VV) participated in target delineation for this study. For the purpose of delineation, daily MVCT scans for three patients with oropharynx, nasopharynx, and larynx primary tumors treated on helical tomotherapy (HT) were selected. The characteristics of the patients are given in Table 1. For each patient, MVCT imaging was done daily during the course of treatment, but for the purposes of our evaluation image studies from each week of therapy were used. A total of 34 studies, 17 original and 17 enhanced, were available for analysis. Both the original and enhanced MVCT sets were transferred to the Pinnacle treatment planning system (Pinnacle^3^ version 8.0d; Philips, Fitchburg, WI). For the three patients, each physician was asked to delineate the gross tumor volume (GTV) on both original and enhanced MVCT studies pertaining to each week of treatment. MVCT images are primarily used for image registration with kVCT planning studies, but because they are also required for plan adaptation and in some cases (e.g., double hip prostheses) initial plan is done with only MVCT set, physicians were not shown patient's kVCT images, as these data could influence contouring decisions and impact interpretation and evaluation of MVCT image quality. Target delineation was performed under no specific guidelines or delineation protocol. All physicians were asked to contour based on their clinical expertise. Participants were not provided with any indicative knowledge as to whether a given image set was enhanced using image enhancement software or not, and no exchange of information pertaining to the contours drawn among physicians was allowed.

**Table 1:** Patient characteristics.

*Patient*	*Age*	*Sex*	*TNM*	*Site*	*Prescription (Gy/Fx)*	*MVCT Datasets*
1	84	M	$T_{4}N_{0}M_{1}$	Larynx	30/10	3
2	70	M	$T_{3}N_{0}M_{0}$	Oropharynx	60/30	8
3	65	F	$T_{2}N_{0}M_{0}$	Nasopharynx	60/30	6

### A. Data analysis

To quantitatively evaluate the variabilities in target delineation between the physicians in a group and by each individual physician between two sets of MVCT studies, two metrics were used. The DICE coefficient is a similarity measure used in information retrieval^(19)^ analogous to volume overlap index used by Wang et al.^(20)^ defined as:

(1) $$DC_{i,p}=\frac{V_{1}\cap V_{2}}{(V_{1}+V_{2})/2}\times 100\%$$

For metrics of each individual physician, the DICE coefficient for comparison of contouring on original and enhanced studies by individual participants $p(p=1,\ldots 5),{\text{DC}}_{p}$, has the GTV delineated on the original MVCT study as $V_{1}$, and the GTV delineated on the enhanced MVCT study by the same physician as $V_{2}$. The DICE coefficient for comparison of contouring by all participants on either original or enhanced MVCT images *i*, ${\text{DC}}_{i}$, is calculated for $V_{1}={\text{GTV}}_{\Pi }$ and $V_{2}={\text{GTV}}_{U}$, where ${\text{GTV}}_{\Pi }$ is the common volume of agreement contoured between all physicians and ${\text{GTV}}_{U}$ is the volume contoured by any physician for a given week on either original or enhanced MVCT images. For ${\text{DC}}_{i,p}$, a value of 0% indicates no spatial overlap, and a value of 100% indicates complete overlap of the delineated volumes.

To detect the variations between physicians of the contoured GTV, the coefficient of variation is defined as:

(2) $$C_{V}=\frac{\sigma }{\mu }\times 100\%$$

The $C_{V}$ allowed us to analyze how well the inter‐fractional variations in tumor volumes were detected by physicians on original and enhanced MVCT studies based on the anatomical changes in patients occurring between weeks.^(21)^ Here, σ and μ are the standard deviation and the mean values, respectively, of the tumor volumes delineated by all physicians in each patient for each weekly study. All data abstraction was done with MIM Software Suit, version 4.2 (MIMvista Corp. Cleveland, OH), with data analysis done in Excel 2003 (Microsoft Corporation, Redmond, WA).

## III. RESULTS

**Figure 1:**
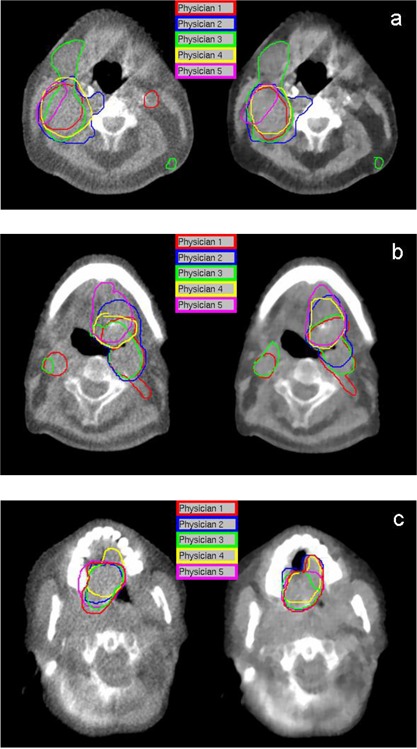
Target volumes delineated by five physicians on (left panels) original and (right panels) enhanced MVCT for: (a) patient 1, week 1; (b) patient 2, week 1; and (c) patient 3, week 1.

**Figure 2:**
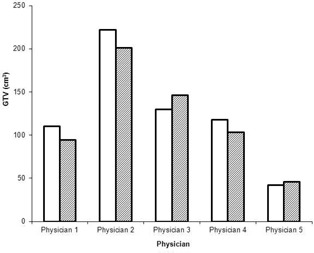
Gross tumor volumes delineated by five physicians on original (solid white) and enhanced (diagonal) MVCT images for the first week of patient 1.

**Figure 3:**
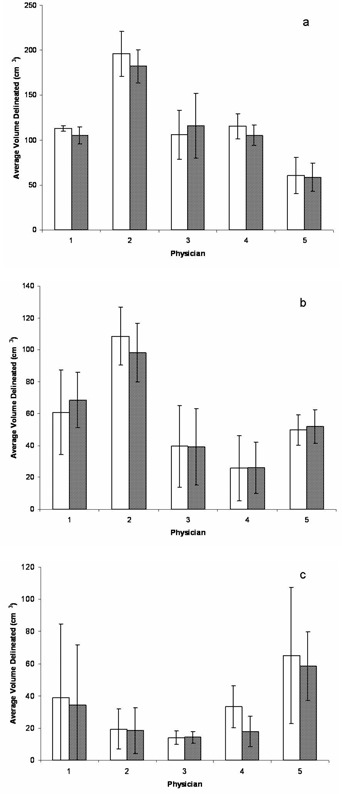
Average target volumes delineated by five physicians on original (solid white) and enhanced (diagonal) MVCT images for: (a) patient 1 over 3 imaging sessions, (b) patient 2 over 8 imaging sessions, and (c) patient 3 over 6 imaging sessions.

A preliminary qualitative assessment of enhanced MVCT images was performed in order to define a set of default enhancement parameters suitable for all observers. Participants viewing the enhanced MVCT screenshots in which contrast alone was varied saw almost unperceivable differences. At higher iteration setting (> 4), fringes began to appear in the enhanced image, resulting in ‘fuzziness’ when contrast was set lower than 50 HU. Similarly, minimal difference was seen at low numbers of iterations when feature granularity was varied (0.5–3.5 cm) for a given noise amplitude. Increasing the feature granularity past 1.5 cm appeared to make no difference at high iterations, whereas decreasing it resulted in ‘blotchiness’. When the iteration number setting was varied for different noise amplitude and feature granularity setting combinations, most participants preferred it set to 4. At lower number of iterations, the enhanced images were almost the same as the original images, while the enhanced images were too blurred when five or more iterations were used. Based on these qualitative results, the following settings were chosen for all enhancements in this study: noise amplitude at 100 HU, feature granularity at 0.5 cm, and an iteration number setting of 4. Van Hoe et al.^(22)^ showed that inadequate window level settings can lead to increased uncertainty in tumor volume delineation; therefore, the window/ level settings for all original and enhanced images were consistent throughout the study. Figure 1 shows target contouring by participants for the first week of imaging on both original and enhanced MVCT images for three patients. Qualitatively, these images show good agreement between physicians in what they believed was the tumor volume. The changes between physicians in target delineation on original and enhanced MVCT images for one week of imaging are illustrated in Fig. 2. To measure variability by individual observer, DICE coefficients $\left({\text{DC}}_{p}\right)$ for targets contoured on original and enhanced MVCT studies were averaged over the weekly imaging sessions for all patients and are presented in Table 2. Average delineated volumes by all five physicians for all patients are shown in Fig. 3, and here we see delineation trends between observers changed on both an image to image and patient to patient basis. DICE coefficients $\left({\text{DC}}_{i}\right)$ for the ${\text{GTV}}_{\Pi }$ and ${\text{GTV}}_{U}$ structures, as well as coefficients of variance between physicians for original and enhanced MVCT for each week are given in Table 3. A matched pair t‐test was performed using the paired percentages for both DICE coefficients and coefficients of variance across the 17 original and enhanced image studies with statistical significance set at *p* ≤ 0.05. While DICE coefficients did not show a statistically significant discrepancy between original and enhanced MVCT (p=0.482), the coefficients of variance showed a statistically significant reduction (p=0.038) between physicians for enhanced MVCT with a 95% confidence interval about the mean difference of (0.5, 13.6).

**Table 2:** Each individual physician's DICE coefficients $\left({\text{DC}}_{p}\right)$ averaged over all imaging sessions.

	*Patient 1* ${\text{DC}}_{p}(\%)$	*Patient 2* ${\text{DC}}_{p}(\%)$	*Patient 3* ${\text{DC}}_{p}(\%)$
Physician 1	82.9±4.6	76.5±15.0	82.2±5.4
Physician 2	82.5±2.3	78.7±7.3	78.4±12.4
Physician 3	79.2±2.5	65.3±5.1	73.0±13.9
Physician 4	76.7±4.3	63.2±7.6	64.0±16.7
Physician 5	80.3±2.8	86.9±3.0	80.8±6.7
Average	80.3±2.5	74.1±9.8	75.7±7.4

**Table 3:** DICE coefficients $\left({\text{DC}}_{i}\right)$ averaged over five physicians, as well as coefficients of variance between physicians for each weekly imaging session for each patient.

		*MVCT*		*Enhanced MVCT*	
*Patient*	*Week*	${\text{DC}}_{i}(\%)$	$C_{V}(\%)$	${\text{DC}}_{i}(\%)$	$C_{V}(\%)$
	1	17.4	51.9	24.9	49.5
	2	46.5	27.4	43.2	31.1
1	3	24.3	47.7	35.5	41.3
	Avg.	29.4	42.3	34.5	40.7
	SD	15.2	13.1	9.2	9.2
	1	12.2	31.0	10.4	29.8
	2	9.2	53.2	5.5	52.8
	3	3.5	64.3	9.2	32.7
	4	0.6	61.6	0.0	69.6
2	5	0.9	61.7	0.3	52.5
	6	9.0	77.2	11.7	55.9
	7	12.2	85.5	13.9	70.4
	8	9.17	81.0	7.9	80.1
	Avg.	7.2	64.4	7.4	55.5
	SD	4.8	17.5	5.1	17.8
	1	15.2	65.3	0.2	76.1
	2	37.7	51.4	32.1	55.2
	3	0.0	71.5	24.1	38.0
3	4	15.6	81.8	14.7	82.6
	5	14.6	95.4	20.3	85.4
	6	9.2	127.1	7.9	112.5
	Avg.	15.4	82.1	16.6	74.9
	SD	12.4	26.6	11.5	25.8

Participating physicians delineated similar GTV contours on both original and enhanced MVCT studies, as demonstrated by the high ${\text{DC}}_{p}$ values in Table 2. The averages of all the DICE coefficients for all the physicians were 80.3%± 2.5%, 74.1%± 9.8%, and 75.7%± 7.4% for patients 1, 2, and 3, respectively.

The ${\text{GTV}}_{\Pi }$ vs. ${\text{GTV}}_{U}$ comparison quantified by ${\text{DC}}_{i}$ allowed us to compare the volume contoured by all physicians to the volume contoured by any given physician in Table 3. Average imaging DICE coefficients $\left({\text{DC}}_{i}\right)$ over five physicians and over all MVCT pairs included in this study were higher for enhanced MVCT, more pronounced for patient 1 (34.5% vs. 29.4%) than for patients 2 (7.4% vs. 7.2%) and 3 (16.6% vs. 15.4%).

## IV. DISCUSSION

This study is the first on the reproducibility of GTV delineation comparing original and software enhanced MVCT images. Patient's MVCT scans are acquired daily prior to treatment on tomotherapy for registration and dose verification calculation purposes. This practice demands the need for images of sufficient quality to ensure dose delivery verification and/or adapted treatment.

Better consistency in contouring on enhanced MVCT studies is also supported by decreased variance between physicians quantified by the coefficient of variance (see $C^{v}$ values in Table 3) on 2/3, 7/8, and 3/6 weeks for patients 1, 2, and 3, respectively. While each physician contoured similarly on both original and enhanced MVCT, their level of agreement was higher and more consistent for enhanced MVCT images. Overall, of 17 pairs of image studies, 12 showed a reduction in delineation variance amongst physicians on enhanced MVCT. Previous studies have stated the difficulties in head and neck tumor delineation. Rasch et al.^(23)^ demonstrated higher variability for head and neck cases than prostate^(24)^ and brain.^(25)^ For head and neck cases, structures are typically not well defined on CT, therefore what physicians consider to be the tumor volume and surrounding healthy tissue can be highly variable. According to Weltens et al.,^(26)^ this uncertainty is related to the experience of the contouring physician, as well as each individual's knowledge of normal and radiological pathology. Unlike previous delineation studies where single image sets are contoured for multiple patients, this study consists of multiple image sets for individual patients and as such, differing perceptions in contouring patient volumes emerged. Some participating physicians consistently contoured the same volumes throughout the course of treatment for all patient images in a conservative manner, while others adapted to the new set of enhanced images and were less conservative about the tumor volume they delineated. There is an advantage to enhanced MVCT in terms of increasing physician agreement when contouring the GTV for the selected cases in this study. More importantly, the process of image enhancement allows us to acquire improved image sets without adding dose to the patient.

The limitations of this study are similar to those of previous delineation studies with regards to the clinical target volume (CTV) and planning target volume (PTV).^(27)^ Only the GTV was contoured by the participating physicians. Delineation of the CTV and PTV is more complex, as different physicians may consider using different margins for construction of the CTV and PTV. Welten et al.^(26)^ also stated that variation in the delineation of the GTV subsequently increases the uncertainty in the CTV and PTV. Recent studies have combined CT imaging with other modalities such as magnetic resonance imaging (MRI)^(28)^ and positron emission tomography (PET)^(29)^ in hope of improving GTV delineation accuracy. By reducing the variability and uncertainty associated with GTV delineation on raw MVCT, the possibility of combination with other imaging technologies may lead to more accurate CTV and PTV delineation and improved hybrid imaging strategies, thereby widening the breadth of diagnostic tools used in image‐guided radiation therapy.

## V. CONCLUSIONS

This study has shown that enhanced MVCT provides advantages in terms of reducing variance between physicians in delineation of the GTV. The image enhancement was used over the course of patient treatment regimens and the MVCT image enhancement software provided increased agreement between physicians for all patients.

Future work focusing on different anatomies is needed to determine the ideal settings and uses for image MVCT image enhancement to increase the accuracy of image‐guided radiation therapy techniques. This approach to analysis of image enhancement technique may also be useful for other image acquisition methods to address the issue of target delineation in radiation therapy.
